# Politics of Prevention: Reflections From the COVID-19 Pandemic

**DOI:** 10.1177/2632077020938360

**Published:** 2020-07

**Authors:** John L. Romano

**Affiliations:** 1 University of Minnesota, Minneapolis, MN, USA

**Keywords:** COVID-19, prevention, prevention science, psychology, politics

## Abstract

The COVID-19 pandemic from a prevention science perspective, including research topics, is discussed. Political considerations that influence prevention activities, with examples from the pandemic and from more typical prevention initiatives in schools and communities, are presented. The definitions of prevention science and prevention interventions are delineated, and a brief summary of prevention history is given. The relationship between health disparities and COVID-19 is discussed. Two theoretical perspectives that may help to inform effectiveness of COVID-19 prevention measures, health belief model and theory of reasoned action and planned behavior, are summarized. This article emphasizes the importance of adapting prevention applications to the intended recipients, especially ethnic and cultural groups. The need to strengthen prevention training in graduate education and strategies to reform the education to meet accreditation and licensing standards are suggested.

Daily news reports, social media messages, press conferences, and other sources provide information and opinions about the coronavirus pandemic that has swept across the world like a major natural disaster. However, unlike natural disasters, the virus, also called COVID-19, the disease caused by the novel virus, knows no boundaries, and at this writing, neither a vaccine nor therapy has been developed to control the virus. Furthermore, despite months of study by expert specialists across the global scientific landscape, much is unknown about the virus; although, hopefully, more will be understood by the time this article is published. Thus, as of now, prevention is the cornerstone and main strategy to control and mitigate the spread of the virus. Although COVID-19 research has been initiated among social scientists, the research projects this author has seen focus on the important psychosocial effects of the virus, such as managing anxiety and stress, and providing psychological support. This author, appreciating that his sources are limited, has yet to see a social science research project that studies the effectiveness of recommended prevention interventions or other virus prevention initiatives from a psychosocial perspective. The National Institute of Mental Health (2020) recently published its strategic plan for research with prevention and cure as one of its major goals. It is timely to mount interdisciplinary research projects that address the psycho–social–behavioral aspects of COVID-19 prevention recommendations and other initiatives. Therefore, it is appropriate that the inaugural issue of the *Journal of Prevention and Health Promotion* (*JPHP*) includes a paper that speaks to this historic global pandemic, which relies primarily on prevention science and prevention interventions to reduce illness and death caused by COVID-19.

Prevention is an interdisciplinary science, with contributions from many specialties. However, my primary area of training and specialization is prevention psychology. Therefore, I am writing this article from a prevention psychology perspective and recognize that other specialists may offer differing and complementary perspectives.

The article is organized in five sections. Initially, distinctions between prevention science and prevention interventions are reviewed, along with a brief history of prevention. This discussion is followed by the influence of political considerations on prevention interventions, whether smaller scale interventions or major global interventions recommended to contain COVID-19. In the health disparities, prevention, and COVID-19 section, U.S. population health and economic disparities exposed by the pandemic and their influence on COVID-19 prevention recommendations are highlighted. The section prevention applications: understanding the audience provides guidance for the development of prevention applications. In this section, two theories are summarized: health belief model (HBM; Hochbaum, 1958; Rosenstock, 1974) and theory of reasoned action and planned behavior (TRAPB; Ajzen, 1991; Fishbein, 1967). They are presented as examples of theories with long histories studying prevention interventions and relevant within a COVID-19 prevention context. The future directions: implementing a prevention agenda for applied psychology section of the article offers suggestions for prevention research related to the pandemic, and recommendations for training in prevention science including multidisciplinary education in applied psychology. Throughout the article, examples as they apply to COVID-19 prevention interventions are discussed, as well as prevention projects that might be implemented within local institutions and communities.

## Prevention Science and Prevention Interventions

Prevention science is an interdisciplinary specialization that draws expertise from multiple disciplines, including psychology, social work, medicine, public health, economics, and public policy. The Society for Prevention Research states that the major goal of prevention science “is to improve public health by identifying malleable risk and protective factors, assessing the efficacy and effectiveness of preventive interventions and identifying optimal means for dissemination and diffusion” (Society for Prevention Research, 2011, p. 3). This goal encompasses a broad range of human ecology across the life span and, within various environments, whether they be schools, communities, or nations, to maximize health and well-being.

Prevention science is the foundation for the development of prevention interventions. Early on, Caplan (1964) developed a now classic framework to categorize prevention interventions. Caplan called prevention interventions (a) primary (to prevent a disease or illness and suitable for everyone, such as mass media vaccination messages), (b) secondary (delivered to those at risk, such as teen sex education programs), and (c) tertiary (to reduce the impact of an existing problem, e.g., rehabilitation programs for stroke victims). Caplan’s framework was initially designed for public health or medical preventive interventions, such as childhood vaccinations, although the framework has been regularly applied to social, emotional, and behavioral interventions. However, in the context of behavioral health, primary prevention may not be a goal as preferred behaviors may change at different periods of a person’s life. For example, a school-based prevention intervention goal might be to reduce teen pregnancy or delay alcohol use through psychoeducational interventions, but these will change as the adolescent matures into adulthood.

As a follow-up to Caplan (1964), Gordon (1987) presented a continuum of prevention interventions that he labeled (a) universal, (b) selective, (c) indicated. Universal interventions, like primary prevention, are for everyone within a population or targeted group. Selective and indicated interventions (like secondary prevention) are designed for those at lesser or greater levels of risk in relation to the problem or disorder. Gordon did not believe that tertiary interventions belonged within a prevention intervention classification scheme because the problem had already occurred. Gordon’s intervention classification was adopted by the Institute of Medicine’s Committee on Prevention of Mental Disorders (Mrazek & Haggerty, 1994).

More than 20 years ago, Romano and Hage (2000) expanded on earlier categories of prevention interventions presented by Caplan (1964) and Gordon (1987) to include the promotion of individual protective attitudes, behaviors and skills (protective factors), and systemic and advocacy interventions to promote health and well-being. Others have also expanded prevention interventions to include promotion of protective factors (Conyne, 2004; Cowen, 2000; National Research Council and Institute of Medicine, 2009) and advocacy for systemic interventions that promote community health (Pieterse et al., 2013; Prilleltensky, 2001).

In terms of individuals and communities, promotion of protective interventions might include, for example, strengthening family-based services, offering affordable and quality child care services, providing community parent education programs, conducting workshops on job-seeking strategies, and promoting increased community adolescent recreational opportunities. Numerous examples have been implemented in schools for many years, including social–emotional learning programs designed to foster healthy peer relationships, self-awareness, and enhance self-esteem.

Since mid-March 2020, U.S. public health professionals have strongly recommended practices to protect citizens from COVID-19. Very quickly, most citizens know about the potentially lifesaving behaviors, for example, stay at home and maintain social distance when outside the home, frequent handwashing, and masks in public. These behaviors of lifestyle rapidly became very common for most people across the globe. It is ironic that given the tremendous advances in medicine and other fields during the last 100 years, as of now, these protective preventive interventions are the best tools to contain the spread of the virus. Studies of COVID-19 preventive interventions offer rich potential to prevention scientists, researching topics such as effectiveness of recommended behaviors, compliance across different demographic groups, and effectiveness of varying media messages.

Systemic prevention interventions that enhance personal, social, and physical well-being across institutions, communities, and larger entities, such as cities, states, or countries, have been advocated across many different problem areas (American Psychological Association [APA], 2014). For example, tobacco use and secondhand exposure is a major health hazard. As a result, amid much controversy, many communities across the United States and beyond prohibit the use of tobacco products in bars, restaurants, and other public places, such as outdoor recreational areas. To reduce addiction risk among teenagers and young adults, communities have also enacted preventive legislation by increasing to 21 years the legal age to purchase tobacco products.

Another systemic intervention example is the restrictions on the marketing and purchasing of vaping products and e-cigarettes as communities have moved quickly to control advertising and purchases. The Centers for Disease Control and Prevention (CDC, n.d-b) has put forth strong recommendations against their use, considering them unsafe for youth, young adults, pregnant women, and adults who are not using tobacco products. Furthermore, although they may have some benefits to help tobacco users stop using tobacco, the health risks are unknown as is their ability to assist in smoking cessation. As such, e-cigarettes and vaping have been heavily regulated or banned in many countries and in several U.S. states (CDC, 2019; Global Center for Good Governance in Tobacco Control, 2019).

Several years ago, South Korea initiated a country-wide initiative to prevent internet addiction (Cho, 2017). The systemic intervention includes several components delivered across the population, including addiction prevention education in schools, training internet addiction counselors, and comprehensive social media campaigns. In the United States, an ongoing and contentious battle on gun control and gun availability has been waged over many years (Spitzer, 2016), and the American Public Health Association calls gun violence an epidemic (Benjamin, 2015). Many scholars and prevention specialists argue that stricter gun-control measures save lives, whereas opponent objections are based on the second amendment of the U.S. Constitution (right to bear arms) and restriction of individual freedoms.

In the United States, and other countries, many systemic prevention strategies are recommended and, in some cases, required, in attempts to mitigate the spread of COVID-19. Several states instituted “stay at home” policies and other recommendations. However, these measures have resulted in a severe economic depression across the country. The economic consequences have created a vigorous debate about the necessity for the prevention recommendations in parts of the United States. Although legislation has provided some financial compensation for businesses, and unemployment benefits for employees, the effects of the economic decline are devastating for many in the United States. The debate is a reminder that political considerations are very important to address when designing prevention interventions.

## Politics of Prevention

Political considerations can influence the level of support for preventive actions. Therefore, it is important that prevention specialists consider the political dynamics that may surround a prevention intervention proposal, whether on a small scale as in one school, or a large school district or community. Although prevention specialists will be excited about an intervention they wish to implement, they must be cognizant of the political dynamics that surround an intervention. Therefore, it is necessary for prevention specialists to carefully assess sources of support for and resistance to an intervention. An intervention that is well supported in one locale or group may lack support in another group or setting. Careful attention to communicating with key stakeholders at the earliest stages of a prevention project is critical.

As the COVID-19 pandemic has unfolded, preventive recommendations to reduce the virus spread have exposed major differences among stakeholders, regions, and political beliefs. The differences include social distancing and face mask use recommendations and timelines to open businesses, gatherings for religious purposes, and recreational areas. The core controversies center around economic issues, citizen health and well-being, and individual freedom versus the common good. Specialists from fields such as medicine and public health, and government officials debate the urgency and actions needed. The differences have become more disparate as the pandemic has evolved. Some become impatient with prevention recommendations as they impinge on personal freedoms and reduce sources of financial and social support and pleasure. Of course, political disagreements surrounding the prevention of the COVID-19 virus are much greater and immediate threats to health and well-being compared with more typical prevention applications that specialists offer in schools, communities, and workplaces. However, knowing about and considering differences among stakeholders are critically important for the success and sustainability of a prevention project.

As an example, instructive for this discussion with relevance to prevention and psychology, is the process to gain APA approval of the *Guidelines for Prevention in Psychology* (APA, 2014). The *Guidelines* were approved by APA Council after about 5 years of development by a *Guidelines* Task Force of APA members. Although there were obstacles during the journey to approval, one is especially important in the context of this article. *Guidelines* drafts were reviewed by APA Committees and Boards as well as stakeholders within the public domain (e.g., state boards of psychology). One of the major concerns of APA governance bodies during the review process was the inclusion of phrases and terms such as “social action” and “advocacy.” According to APA governance at the time, guidelines are not designed to promote a social agenda. Thus, to proceed with the approval process, the Task Force made concessions to remove these terms from the title and body of the article. Interestingly, APA has a very active advocacy initiative within its structure, reporting regularly to the membership about its work with policy makers on topics such as promoting social justice and human rights, reducing health disparities, addressing violence prevention, and encouraging members to do likewise. Perhaps APA only objected to the inclusion of the terms in guideline development at the time of approval, and the policy has now changed. However, at the time, the *Guidelines* Task Force was surprised by the APA position, because much prevention activity is focused on advocacy and social justice (Kenny et al., 2009; Kenny & Hage, 2009; Romano, 2015). Although the *Guidelines* were eventually approved, APA concerns about terminology and language were unexpected and caused significant delays in eventual approval.

Just about everyone agrees that “prevention is better than cure.” However, prevention specialists, especially those newer to the field, would be wise to consider differences among recipients and stakeholders. The implementation of prevention projects will often be supported or resisted in ways that mirror the larger population in which the prevention project is implemented. Furthermore, as seen with COVID-19 prevention recommendations, recipients and stakeholders may lose patience with prevention interventions as outcome evaluations do not yield immediate results. Although other types of evaluations (e.g., formative) are useful, stakeholders (e.g., community leaders, political figures) may expect an intervention to correct a problem rapidly. However, as seen with the hurried attention to develop a COVID-19 vaccine, infectious disease scientists remind us that development will take considerable time, require collaboration across the scientific community, and incur considerable costs before its effectiveness and safety can be established (Corey et al., 2020). Of course, developing a vaccine for a worldwide pandemic does not compare with local psychosocial prevention interventions, but the development, effectiveness, and sustainability of an intervention is, nevertheless, demanding and time consuming.

In an APA convention presentation (Romano, 2013), I discussed three issues, not mutually exclusive, that are likely to lend controversy to prevention interventions, even though, at the outset, all might agree that the prevention idea is good. The issues are (a) values, (b) morality, and (c) economics. First, understanding individual and community values related to potential prevention interventions is important. A value-related issue is differences between the needs of the individual and needs of the community. What is good for the community may not be supported by individuals. In highly individualistic cultures such as much of the United States, collectivistic beliefs will create controversy. In the COVID-19 pandemic, recommendations to practice social distance, stay-at-home, and face masks, as measures to protect community health, have been resisted and angrily protested in U.S. cities. The issue is complex due to differing values between individuals, communities, and regions of the United States. Furthermore, due to work requirements and socioeconomic levels, some do not have the luxury of staying at home (e.g., health care providers, grocery store employees). Brown (2020) comments that stay-at-home and social distance recommendations are choices available to wealthier members of society, less so for members of lower socioeconomic groups.

In collectivistic societies, with values and behaviors associated with community benefits, rather than individuals, citizens are more accepting of country-wide policies that have the potential to reduce community spread of COVID-19. Drawing comparisons between countries is difficult, due to factors such as enforcement of preventive regulations, availability of virus testing, methods of reporting, and population density. However, a few examples are illustrative. As of May 16, 2020, the United States had 4,526 COVID-19 cases and 269 deaths per 1 million population, whereas South Korea had 215 COVID-19 cases and five deaths per 1 million population, Singapore had 4,681 COVID-19 cases and four deaths per 1 million population, and Malaysia had 213 COVID-19 cases and 3 deaths per 1 million population (Worldometer, 2020). All three Asian countries, with a tradition of collectivism, have much lower death rates compared with the United States. Although Singapore’s incidence rate is like the United States, the other two countries have much lower incidence rates compared with the United States.

Values are also related to the use of contact tracing, a prevention strategy used by public health professionals to mitigate spread of community disease. Contact tracing is a process of contacting individuals who have been in close contact with someone who tested positive for the virus to recommend self-quarantine. Contact tracing is used in different countries and the United States. However, the strategy offers disadvantages, including training of public health personnel who are not familiar with contact tracing, costs, reluctance of people to accept information when notified that they have been exposed to the virus, and resistance of citizens to submit to government surveillance (Temple, 2020). The last disadvantage will be especially prominent if widespread surveillance is conducted via cell phone apps. Citizens in more individualistic countries are more likely to resist what they perceive as threats to freedom and privacy, and governmental interference. Singapore has been using contact tracing via cell phone apps since March 2020, perhaps one reason for the country’s low COVID-19 death rate. The United Kingdom is developing a similar cell phone plan as a strategy to more quickly reduce virus spread and open the country to increased freedom of movement (Chowdbury et al., 2020).

Values influencing prevention interventions were also revealed in the debate about cigarette smoking. In some locales, tobacco use is prohibited in closed spaces, and some cities also prohibit tobacco use in outdoor areas. Tobacco use regulations vary across U.S. communities. Similarly, in the context of schools, differing values among educators about the amount of time children are excused from academic classes to participate in social–emotional learning activities requires discussion. Prevention specialists need to work with educators and parents to balance academic instruction with proposed psychosocial prevention activities to reduce resistance to the intervention. Methods to resolve differences will be different based on school subjects, student grade level, school administrators, and parental preferences.

The second issue to consider in prevention intervention planning is morality. An example from the COVID-19 pandemic is the issue of attendance at religious ceremonies and events when stay-at-home and social distancing orders are in place. Some argue that during this time of distress and need for community, it is especially important that people congregate with members of their faith community. Others contend that following the stay-at-home recommendation is the more moral position to stay healthy and minimize the virus spread. In a school-based example, some parents will accept and deem important prevention programs that teach sex education to develop healthy sexual behavior, reduce teen pregnancy, and promote respect and acceptance of different sexual identities. Other parents will disagree, stating that this type of education is best left to parents and the family. Also, bully prevention programs in schools generally receive strong support. A component of such programs to indirectly reduce bullying behaviors might include promotion of social groups and increased mental health support of students who are more likely to be bullied (e.g., lesbian, gay, bisexual, transgender, and queer [LGBTQ] students, special needs students). The need for such interventions is best explained to parents and stakeholders who may not be fully aware of the importance of the intervention in a comprehensive bully prevention program.

The third issue that merits discussion is the economics of prevention. Finances may be a more acceptable form of resistance and used to camouflage other reasons for resisting, “this is a good idea, but we just can’t afford it.” This argument has been used in the COVID-19 pandemic as local and national leaders debate the importance of relaxing stay-at-home recommendations to support local businesses and community economies. Similarly, communities in the United States have outlawed the sale of electronic vaping devices to anyone below 21 years. Cities have instituted such laws based on the potential harmful effects of vaping and danger of nicotine addiction, especially in brain development of adolescents. However, stores that sell these products may lose business, similarly, to bans on selling tobacco products to adolescents and young adults.

Another economic issue relates to the mental health of youth and young adults. Specifically, the need for mental health services for children, adolescents, and postsecondary students is growing rapidly, and resources to serve students in educational institutions are inadequate (Hunt & Eisenberg, 2010; Kaffenberger & O’Rouke-Trigiani, 2013; Oswalt et al., 2020). The units that house school counselors, school social workers, college and university counselors, and psychologists are often understaffed in educational institutions. Mental health professionals are heavily engaged in crisis-intervention work, which leaves less time for prevention activity. Data showing school counselor shortages have been presented for many years by the American School Counselor Association (ASCA). ASCA recommends a ratio of one school counselor to 250 students, whereas the mean ratio across the United States is 455 students to each counselor, with a range across the states from a low of 202 to 1 to a high of 905 to 1 (Bray, 2019). Different reasons across the states can account for such large discrepancies, but insufficient funding to support mental health professionals in schools and higher education usually resolves around limited public education funding and differing educational priorities (Leachman et al., 2017; Mitchell et al., 2017).

Recent advocacy for increased student mental health support occurred in the St. Paul, Minnesota, school district when teachers went on strike in March 2020. This was the first district strike in 74 years. One of the main grievances of the educators was lack of student mental health support personnel. The strike ended just before the schools closed due to the pandemic, but not before the district agreed to increased funding for student mental health personnel.

Funding decisions and values are intertwined, as values dictate spending, whether in personal finances, or within a large unit or system. Funds are dispersed based on values, and funding will dictate the strength and scope of prevention initiatives. A disadvantage of many prevention interventions is that immediate results are not usually realized. Therefore, prevention leaders must keep stakeholders engaged in the project through regular reporting of progress and evaluation processes.

A final example that relates to values and funding is the suspension in fall of 2017 of the National Registry of Evidence-Based Programs and Practices (NREBPP) by the U.S. government. In January 2018, NREBPP was no longer funded by the U.S. government. NREBPP was a Substance Abuse Mental Health Services Administration (SAMHSA) program that had evaluated prevention programs across topics and age groups since 1997. Despite objections to the closure of NREBPP from different sectors of the country, federal health officials stated that NREBPP had a flawed system of evaluating programs, and a new system would replace it. The new system, also sponsored by SAMHSA, is called Evidence-Based Practices (EBP) Resource Center. However, Green-Hennessy (2018) stated that NREBPP had a long history, and the system had been strengthened over the years, and rather than replace NREBPP, the money could have been better spent to eliminate weaknesses or flaws in NREBPP. Perhaps there were other motivations for replacing NREBPP, but its demise was shocking to prevention specialists as NREBPP was an important resource. Hopefully, the EBP Resource Center is sufficiently improved compared with NREBPP to justify the funds to create it.

## Health Disparities, Prevention, and COVID-19

As the COVID-19 panic spreads across the United States, vast differences in incidence and death rates within population groups are observed. Although the data are incomplete as most jurisdictions have not reported data by race and ethnicity at this writing, what has been reported is alarming and distressing. For example, news outlets report that African Americans in some of the largest cities account for many more virus incidences and deaths disproportionate to their numbers in the population. Data from Chicago show although people who are Black make up 30% of the city’s population, they account for 68% of the city’s COVID-19 fatalities, and 58% of the virus cases. Similar data were found in Milwaukee, where people who are Black make up 26% of the city’s population, but account for 81% of deaths. Michigan and Louisiana show similar disproportionate data (Cineas, 2020; Johnson & Buford, 2020). Similarly, the CDC (n.d-a) reports New York City data showing virus death rates substantially higher for people who are Black/African Americans and Hispanic/Latinx persons compared with people who are White and people who are Asian. As of mid-April 2020, data show the death rate for people who are Black at 92.3/100,000, Hispanic/Latinx persons at 74.3/100,000, people who are White at 45.2/100.000, and people who are Asian at 34.5/100,000. The devastating impact of the virus on the Navajo Nation populations was reported by Silverman et al. (2020), showing that the Navajo Nation had the highest per capita cases of COVID-19 in the United States at 2,304/100,000 surpassing New York City at 1,806/100,000.

Multiple reasons account for these disparities including U.S. history of racism among ethnic minorities that leads to discrimination, low social economic status, inadequate or lack of health care, limited English language proficiency, immigration status, housing in confined spaces, and homelessness. Furthermore, the pandemic’s universal prevention recommendations are difficult or impractical to follow for many. Frontline (e.g., health care personnel, factory workers, grocery store employees) employee work responsibilities cannot be conducted from a distance, and they are often lower paid. Thus, they do not have the luxury of following stay-at-home recommendations (Brown, 2020).

The COVID-19 pandemic has shed a bright light on health care inequities and disparities in the United States. Health disparities have been a focus of scholars and U.S. officials for some time. The U.S. Office of Disease Prevention and Health Promotion (n.d.) notes that groups within the United States experience health disparities that contribute to poor health and ability to achieve maximum health. Groups include those based on race and ethnicity, sex, sexual identity, age, disability, socioeconomic status, and geographic location. Research from different scholarly perspectives has examined health disparities, including differences between rural and urban areas (James et al., 2017), impact of racial oppression on health outcomes (Gale et al., 2020), public policy solutions to address disparities (Assari, 2018), health care experiences of transgender binary and nonbinary university students (Goldberg et al., 2019), and access to integrated health care (Buki & Selem, 2012; Tucker et al., 2019).

In addition to spotlighting health inequities, COVID-19 has also exposed extreme xenophobia, racial harassment, and discrimination primarily against Asian populations. A few U.S. leaders may have fueled this behavior by referring to the virus as the “Chinese virus,” which some may interpret as people of Chinese ancestry spreading the virus, although leaders have denied the accusation. Although face masks have become more regularly used as the virus has spread across the country, some Asians feel stigmatized by using them, and thus, putting their health at risk (Zhou et al., 2020).

The social, emotional, psychological, and behavioral components of preventing COVID-19 illness and deaths are important areas of study for prevention scientists. However, regardless of whether prevention interventions are large or small, to maximize positive outcomes, the interventions must be culturally relevant and prevention specialists culturally competent, partnering with population groups receiving the prevention intervention (Reese &Vera, 2007). The next section will further expand on this topic.

## Prevention Applications: Understanding the Audience

The above discussion provides examples on how differing values, morality, funding, and ethnic and socioeconomic disparities can influence prevention initiatives, whether they be worldwide and very dangerous pandemics such as COVID-19 or local prevention applications. This section will summarize suggestions to assist prevention personnel as they develop prevention projects, and present them to stakeholders, including policy makers, community groups, and project recipients. It is understood that each stakeholder group may have different opinions about a prevention project, and they are likely influenced by their values, questions of morality, and funding considerations. Therefore, the prevention specialist must be willing to dialogue with members from each of the stakeholder groups prior to initiating an intervention. Some of the dialogue may be informal or in formal group meetings. Prevention activities that seem quite important and necessary to the prevention specialist may not be so for others who will have control over the implementation process, ongoing activities, evaluation, and sustainability of the intervention.

The setting for a prevention intervention can vary from a relatively small institution (e.g., schools) to larger community settings, or, as with the pandemic, a global initiative. In the United States, pandemic media coverage is primarily focused on the United States, but there are implications for other nations in terms of working together to prevent virus spread. For example, nations are restricting air and sea travel across borders, and nations are collaborating on sharing medical supplies and working to develop a therapy and vaccine. However, some of the issues have been contentious and opinions vary on the importance of collaboration across nations and among political leaders. The United States and other nations are operating in unchartered waters with respect to COVID-19 decision making, as the last global pandemic occurred in 1918, when population size, health industries, communication systems, and world dynamics were very different. Countries determine to what extent they will collaborate, either through global organizations, such as World Health Organization, or within regions. Decisions will be driven by values, beliefs, trust, and importance attached to collaborate versus going it alone. Within the United States, several adjoining states have formed collaborations to share knowledge and strengthen the impact of their prevention measures.

Similarly, prevention initiatives on the local level are likely to be successful and sustainable if local leaders, recipients, and beneficiaries of the prevention initiative are consulted from the very beginning of the project. One way to begin the dialogue is the formation of an advisory group. This group is best composed of members who have technical expertise about the project, represent the cultural and demographic characteristics of the community (or school), and are political stakeholders in the community. It is important that one or two coleaders of the group are invested in the success of the project but who have not initiated the project. The advisory group can then begin to discuss the project in relation to community needs and how best to meet the need.

In developing prevention activities, it is recommended to consider not only behavior that needs to be prevented (e.g., school bullying) but also behaviors that are promoted to serve as protections for individuals and the larger community (e.g., respectful and inclusive school environment). Comprehensive prevention projects are best designed to stop or decrease problem behaviors by reducing risk factors, promoting protective factors, and addressing community (school) wide interventions that reduce risks and support protections. Thus, a robust prevention project will emphasize activities that are individual or small group oriented, as well as systemic interventions designed to reduce risks and promote protections across the system whether a school, school district, city, or other entity.

Major COVID-19 prevention recommendations to prevent spread of the disease include stay-at-home, frequent handwashing, maintain social distance, and wear face masks to reduce risk and increase protection for self and others. The guidelines are followed and enforced in varying degrees of consistency within the United States and globally. Citizens decide the best behavior for themselves and the community, not unlike other prevention recommendations (e.g., seasonal flu shot, refrain from tobacco use). Although it took many years for some jurisdictions to approve legislation to restrict cigarette smoking in public places, for example, the highly contagious coronavirus does not allow the luxury of time, and citizens are dependent on public health and political leaders to offer prevention recommendations for the good of society. However, as with other types of prevention recommendations, individuals have freedom of choice to follow them in most countries.

Most prevention specialists will have more modest and less immediate goals compared with stopping a global pandemic. There is a long history of prevention and promotion interventions across institutions and communities such as preventing sexual harassment and abuse on college campuses, reducing gun violence in communities, promoting social–emotional learning in children and youth, ending illegal drug use and inappropriate use of legal drugs across the life cycle, and preventing suicide (Vera, 2013). These problem behaviors are traumatic and potentially deadly. Fortunately, there are examples of prevention programs to reduce or eliminate problems within a given context. SAMSHA’s EBP Resource Center, cited above, is one resource to search for prevention initiatives that have been reviewed and evaluated. However, it is recommended that prevention activities be adjusted or adapted to a location and population, as one set of activities and evaluation tools successful in one locale may not be effective in another context (Romano & Israelashvili, 2020). This recommendation was observed in prevention projects that were developed in different countries, but prevention scientists and specialists adapted the previously developed prevention activities to meet the needs and requirements of their own region or country (Israelashvili & Romano, 2017).

Prevention is an interdisciplinary science, but it is not atheoretical. Prevention activities are best grounded in a theoretical framework that will support the intervention activities and the evaluation process. Some of the more commonly taught theories of psychotherapy for clinical use have formed a theoretical basis for prevention interventions (e.g., cognitive–behavioral; Christensen et al., 2010; Montgomery et al., 2009). Motivational interviewing, with person-centered theory as foundational, has also been used in a variety of prevention interventions (e.g., Strait et al., 2012). Transtheoretical model of behavior change has a long history of use within a prevention framework, especially interventions that address behavioral changes to improve health outcomes (e.g., Prochaska et al., 2009). In the following sections, two theoretical perspectives (i.e., health belief model [HBM] and theory of reasoned action and planned behavior [TRAPB]) will be summarized. These were chosen because of their long history within prevention science, and readers may not be familiar with them.

### Health Belief Model (HBM)

HBM was developed within the U.S. Public Health Service in the 1950s to help understand reasons for people not participating in tuberculous screenings to prevent the illness and promote early disease detection (Hochbaum, 1958; Rosenstock, 1974). The prevention goals of COVID-19 are similar in terms of prevention and disease identification. The HBM researchers found that a person’s beliefs about a disease and need for screening helped to differentiate those who participated in the screening and those who did not. HBM can be applied to COVID-19 and people’s willingness to use prevention measures. According to HBM, four personal health beliefs are predictive of whether a person is likely to adhere to prevention recommendations and participate in screenings. They are (a) perceived susceptibility to the disease, (b) perceived severity of contacting the disease, (c) perceived benefits of participating in the prevention measures, and (d) perceived barriers and disadvantages to participating in prevention activities (Romano, 2015). Much research has been conducted to validate HBM variables in diverse populations in the United States and other countries. Examples of the research projects include willingness of low-income African American women to participate in cancer screenings and promoting behaviors that reduce sexual risks (Champion & Sugg Skinner, 2008).

As applied to preventing COVID-19, HBM offers explanations for behaviors. For example, young adults on Southern beaches likely perceive themselves as less susceptible to the virus, compared with older adults. However, as knowledge about the virus has increased, young and middle-aged adults have also been victims of the disease, although not as severely as older persons. Those who understand and accept the benefits of pandemic prevention recommendations compared with disadvantages will more likely use them. According to the HBM framework, delivering targeted pandemic prevention information to subgroups of citizens based on the four HBM beliefs promises to yield more favorable compliance outcomes.

HBM has value as a theoretical framework for more typical prevention projects, especially related to preventing behaviors that impair health. For example, HBM can be helpful to understand behaviors that place adolescents at risk of sexually transmitted infections, pregnancy, and drug and alcohol use. The four components of HBM can give prevention personnel a framework to better understand resistance to following prevention messages and participating in prevention activities. However, it is important to assess the health beliefs of the group receiving the intervention prior to developing prevention messages and activities.

### Theory of Reasoned Action and Planned Behavior (TRAPB)

Theory of reasoned action (TRA) has a long history, dating back to Fishbein (1967) who developed the theoretical framework to better understand the relationship between personal beliefs, attitudes, and behavior. Several years later, Ajzen (1991) added planned behavior (PB) as an extension of TRA to address the amount of control that individuals believe they have over one or more behaviors. TRAPB is more complex than HBM, as TRAPB addresses several variables that can influence participation in a health promotion or prevention campaign. TRAPB posits that intentions to carry out a desired behavior will be more likely followed if the individual’s attitudes, social norms of those important to the person, and perceived personal control support the desired behavior. The relationships of these variables can be presented symbolically as: *behavior ~ intentions ~ (attitudes + norms + control*; Montaño & Kasprzyk, 2008; Romano, 2015). A major component of the theory is a process called elicitation research. The process involves conducting group interviews of a similar but different sample of future intervention participants to ascertain personal beliefs, attitudes, behavioral intentions, social norms, and perceived control over the desired behavior. Once elicitation data are collected, they will inform intervention activities and messages. The theory is widely used. According to a review of 82 theories used in designing and evaluating interventions to change health-related behaviors informed by social scientists, TRAPB was the second most frequently used theory behind the transtheoretical model of behavior change (Davis et al., 2015). According to Fishbein (2000, as cited in Montaño & Kasprzyk, 2008), the theoretical constructs of the theory have been studied in more than 50 high- and low-income countries.

With respect to the COVID-19 prevention recommendations, TRAPB can help explain people’s willingness to follow recommendations. For example, does a person’s attitude about a prevention recommendation lead to increased use? Do others important to the person follow the prevention guidelines and does the person believe they have control over the behavior? With respect to preventing virus spread, most people have personal control over the CDC prevention recommendations, unless employment requirements reduce their assessment of personal control. Also, their intention to follow recommendations is a function of their attitudes toward the behavior and the level of perceived social support to follow the recommendations. For example, in the United States, some leaders are less likely to follow some of the recommendations, resulting in poor modeling and weakening social support for them.

Romano and Netland (2008) describe a hypothetical example of TRAPB. In their example, the authors show how TRAPB and elicitation research are used to reduce physical aggression among sixth-grade boys. Through elicitation research, prevention personnel learn about differences between subgroups of all sixth-grade boys in the school, as it cannot be assumed that all sixth-grade boys (or any group) will have similar beliefs, social support, and perceived personal control to carry out intended behaviors. Without collecting subgroup information about these variables beforehand, differences between subgroups are unknown. Elicitation research provides a process to adjust or better align prevention activities with TRAPB variables important to subgroups, leading to better outcomes.

Of course, other theoretical frameworks to guide prevention projects can be considered by prevention specialists. For example, Conyne (2004) has summarized several prevention strategies, including self-competency facilitation, community organizing and systems intervention, and redesign of the physical environment. If a project is based on a theoretical model, project goals, design, activities, and evaluation methods will help to explain outcomes, and hopefully lead to sustainability as future changes to improve the intervention are made based on the theoretical model.

## Future Directions: Implementing a Prevention Agenda for Applied Psychology

Prevention scientists and applied prevention specialists are experiencing a global epidemic of historic proportions. Prevention is the main strategy to prevent the spread of COVID-19. However, despite overwhelming news coverage and mass media reports, little, if any, coverage is presented on the role of behavioral science expertise in helping to control the pandemic. There are many behavioral science specialists devoted to assisting others in this time of crisis. This activity is highly valued and understandable given the emotional impact of the pandemic. In addition, remediation and crisis-intervention education is prominent within the helping professions. Furthermore, the public’s perception of applied psychology and other helping professions is to fix problems, rather than prevent them. However, prevention science can be instrumental in assisting in multiple ways during this epidemic. For example, prevention specialists from across disciplines and in research teams are well positioned to study prevention-based research questions. Hopefully, some of the research has begun, and the National Institute of Mental Health (NIMH) prevention research agenda cited above will encourage development of future research projects. A few research questions to consider are as follows: (a) Do the major media messages of social distancing, handwashing, and mask wearing serve all segments of the U.S. population equally? (b) How might these messages be perceived within different ethnic, cultural, and socioeconomic groups? (c) What types of media are most effective to reach diverse population groups? (d) How might the health beliefs of different groups influence their adherence to preventive actions? (e) How do attitudes, beliefs, and sense of personal control influence adherence to prevention recommendations? (f) What social influences are most effective to promote the use of prevention recommendations within groups, whether they be family, government officials, or others within personal networks? (f) How does compliance with prevention recommendations compare across nations? These are a few of the questions that can be examined utilizing the expertise of prevention social scientists. It is critically important that professionals from diverse specialties such as psychology, public health, medicine, social work, public policy, and economics work in collaboration in efforts to contain the spread of COVID-19 through preventive measures.

As with other specialties, applied psychology must continue to emphasize and encourage the role of prevention within the profession. For example, in counseling psychology, much has been accomplished, including the publication of this inaugural journal issue. However, much more needs to be accomplished during the next decade, and, hopefully, a more robust recognition of the importance of prevention psychology in the public domain and policy decisions will occur.

### Prevention Training, Accreditation, Licensing

The advancement and prominence of prevention psychology, along with prevention science in other social science disciplines, will require adjustments in training strategies to meet accreditation and licensing requirements. Unfortunately, prevention education is seriously lacking in much of applied psychology, although some progress has been made in the last decade (see Hage et al., 2007; Romano, 2015). As Conyne et al. (2008) discuss, there are multiple ways to provide prevention training within graduate education and postgraduate training. One key component to prevention education is encouraging student coursework outside the major area of study. Applied psychology programs are encouraged to make it more possible for students to enroll in courses in fields such as public health, medicine, social work, public policy, and economics. Furthermore, field work, practicum, and internship experiences could also give attention to training experiences in prevention science. This model of multidisciplinary education can also be more widely applied in other disciplines.

However, graduate programs in applied psychology are already packed with courses to meet accreditation and licensing requirements, but the APA accreditation process may offer some enlightenment. APA is reviewing accreditation standards for the newly developed master’s program in health services psychology (MPHSP; Grus, 2019). My cursory review of the proposed accreditation standards for MPHSP found them lacking in prevention content. Accreditation standards for this program, like doctoral programs, are categorized into broad psychological content areas, and graduate programs usually offer specific courses to meet the standards. Because prevention science education is relevant to multiple content areas (e.g., social, affective, cognitive, behavioral), prevention education can be infused across multiple courses instead of one or more stand-alone courses. This strategy would reduce expansion of the curriculum. If graduate programs show that specific courses or multiple courses that include prevention content meet accreditation and licensing board standards, infusion of prevention education is possible. However, such changes require faculty with interest, expertise, and commitment to prevention science, and students who desire such education.

The COVID-19 pandemic has highlighted the importance of prevention to reduce disease and death. Although it is hoped that COVID-19 is a once-in-a-lifetime pandemic, there will be other epidemics that risk health, hopefully on a smaller scale, and the expertise of prevention scientists from the behavioral sciences will be sought. However, apart from health-related epidemics, prevention science must continue to provide guidance and expertise related to major social problems (e.g., bullying and social violence, poor school achievement, drug and alcohol addiction, racial stereotyping, and sexual harassment). This article highlighted the role of prevention science in COVID-19 while providing examples and applications across schools and communities.

As a final comment, counseling psychology is commended and congratulated for producing this inaugural *JPHP*, only the second journal sponsored by the Society of Counseling Psychology (APA Division 17) in its 75-year history. The journal is an important outlet to disseminate prevention research and scholarship by scientists and practitioners from different disciplines and specialties. It took several years to launch *JPHP*, and now the inaugural issue is published during a massive and deadly global pandemic in which prevention is central to containment of the virus. Appropriately, *JPHP* is being launched at a momentous time in the history of the world.
